# Reproductive autonomy and contraceptive use among women in Hanoi, Vietnam

**DOI:** 10.1016/j.conx.2019.100011

**Published:** 2019-10-05

**Authors:** Nghia Nguyen, Jessica Londeree, Linh H Nguyen, Dung H Tran, Maria F. Gallo

**Affiliations:** a Department of Obstetrics and Gynecology, Vinmec International Hospital, 458 Minh Khai, Hanoi, Vietnam; b The Ohio State University, College of Public Health, Division of Epidemiology, Cunz Hall, 1841 Neil Avenue, Columbus, OH, 43210, USA; c Department of Training and Research, Hanoi Obstetrics and Gynecology Hospital, Lathanh Road, Hanoi, Vietnam

**Keywords:** Contraception, Unprotected sex, Reproductive autonomy, Measurement, Vietnam

## Abstract

**Objective:**

Reproductive autonomy (i.e., power to control and decide about contraceptive use, pregnancy and childbearing) could determine a woman’s capacity to use contraception. Although the Reproductive Autonomy Scale was developed to quantitatively assess women’s reproductive autonomy, it has not been validated in any population outside the United States.

**Study design:**

We conducted a cross-sectional study of reproductive-age, sexually active women in Hanoi, Vietnam, who did not desire pregnancy. We administered a questionnaire containing the Reproductive Autonomy Scale and calculated composite scores of the measure’s three subscales: (1) decision-making power, (2) freedom from coercion and (3) communication ability. To assess internal consistency, we calculated Cronbach’s alpha score for each subscale. We used logistic regression to evaluate differences in subscale scores between women who did and did not engage in unprotected sex in the past month.

**Results:**

Analysis is based on 500 participants; of these women, 17% (*n* = 85) engaged in unprotected sex in the past month. Subscales had moderate to high internal consistency (Cronbach’s alpha: 0.65–0.87). Mean subscale scores did not vary between women who did and did not engage in recent unprotected sex. Unprotected sex in the past month was not associated with decision-making power (adjusted odds ratio [aOR], 0.77; 95% confidence interval [CI], 0.49–1.20), freedom from coercion (aOR, 0.94; 95% CI, 0.52–1.67) or communication ability (aOR, 1.69; 95% CI, 0.92–3.09).

**Conclusion:**

Findings highlight the need to develop and validate a new measure for reproductive autonomy for populations outside the United States or to adapt the existing measure for these contexts.

## 1 Introduction

An estimated 44% of pregnancies worldwide were unintended in 2000–2014 [1]. For women and their families, the consequences of unintended pregnancies are often lasting and severe; unintended pregnancies can lead to poorer health among children, lost educational opportunities and increased levels of pregnancy-related morbidity and mortality [[2], [3], [4]]. Although modern methods of contraception are widely available and effective at preventing unintended pregnancy, a large gap remains between the need for contraception and its use. Of the 85 million women who have an unintended pregnancy every year, 80% are not using contraception at the time of conception [4]. In low- and middle-income countries, where the vast majority of unintended pregnancies occur [1,4], understanding the barriers to contraceptive use among women who want to avoid pregnancy is crucial.

Studies assessing the individual-level factors affecting contraceptive use are often grounded in the assumption that individuals have control over their contraceptive behavior [5]. However, as with other sexual risk outcomes, a woman’s capacity to act upon her intention to use contraception may be contingent upon the wishes and actions of her partner or other members of her family or community. Together with other demographic, social and cognitive constraints to contraceptive use, reproductive autonomy — defined as power to control and decide about matters concerning contraceptive use, pregnancy and childbearing — could determine a woman’s capacity to use contraception [6].

Reproductive autonomy can fluctuate within different relationships and cultural contexts, depending on the degree to which the partner or surrounding community supports reproductive rights [6]. The role of reproductive autonomy on contraceptive behaviors has been documented in several qualitative studies; in contexts where women are disempowered, partner pressure and disapproval [[7], [8], [9], [10], [11], [12]], and poor communication with one’s partner [13] can hinder contraceptive use and acceptance. An important facet of reproductive autonomy known as reproductive coercion (i.e., interference with reproductive autonomy by another party, such as an intimate partner or parent-in-law, though contraception sabotage, pregnancy coercion or other actions to control pregnancy outcome), has also been found to increase the risk of unintended pregnancy [14,15]. While these findings support the role of reproductive autonomy in explaining poor contraceptive use, few studies have quantitatively assessed the role of reproductive autonomy on contraceptive behaviors.

The Reproductive Autonomy Scale was recently developed to quantitatively measure a women’s capacity to achieve her reproductive intentions [6]. This scale represents reproductive autonomy using three constructs: (1) decision-making power (i.e., having primary say — either alone or with a partner — in matters relating to contraceptive use, pregnancy and childbearing), (2) freedom from reproductive coercion and (3) communication ability (i.e., feeling comfortable talking with one’s partner regarding such matters) [6]. Upadhyay et al. validated the scale among 1892 women at family planning and abortion facilities in the United States by demonstrating an inverse association between the freedom from coercion and communication subscales and the occurrence of sex unprotected by contraception in the past 3 months [6].

To date, no study has validated the Reproductive Autonomy Scale in a population outside of the United States. By studying reproductive autonomy within these contexts, we can better identify and understand the influence of interpersonal power on reproductive behaviors, which then can inform strategies to prevent unintended pregnancy. Thus, our objective was to validate the Reproductive Autonomy Scale among a population of women in Hanoi, Vietnam, by evaluating its association with recent engagement in unprotected sex.

## 2 Material and methods

We conducted a secondary analysis of a cross-sectional study of 500 women attending the obstetrics-gynecology department of a large public hospital in Hanoi, Vietnam, in November 2017 to September 2018. The parent study’s objective was to evaluate a novel method to measure women’s implicit beliefs about the safety and naturalness of contraception; these findings will be reported elsewhere. To participate in the parent study, women had to be of reproductive age (18—45 years), have at least a minimal level of literacy, report being comfortable using a computer, be sexually active (defined as ≥ 1 penile—vaginal act in past month), not pregnant or breastfeeding and not want a pregnancy within the next 12 months. Female study interviewers recruited women as they waited for routine care until they enrolled a stratified sample of women who were current users of the intrauterine device (IUD) (*n* = 128), oral contraception (*n* = 126) or neither method (*n* = 239). Participants provided written consent before enrollment, and the institutional review boards at The Ohio State University and the Hanoi School of Public Health approved the research.

We administered a questionnaire on demographics and contraception-related beliefs and behaviors to participants using the electronic data capture tool REDCap. The questionnaire included the 14 questions from the Reproductive Autonomy Scale, which measures three subscales of reproductive autonomy: decision-making power, freedom from coercion and communication ability [6]. The four questions of the decision-making power subscale asked respondents to identify the person who has the final say in decision making [“my partner (or someone else), me and my partner (or someone else) equally, or me”] in various reproductive situations (e.g., whether the woman uses contraception). The other two subscales ask respondents about their agreement (“strongly disagree, disagree, agree, strongly agree”) with 10 statements. The following is an example of a statement used for the freedom from coercion subscale: “My partner has stopped me from using a method to prevent pregnancy when I wanted to use one.” An example of a statement used for the communication subscale is as follows: “My partner would support me if I wanted to use a method to prevent pregnancy.” We generated composite scores for each of the subscales of reproductive autonomy by calculating the mean response to each subset of questions. Higher mean scores indicated greater reproductive autonomy. The questionnaire also assessed various demographic characteristics, including age, education, income and place of residence. We dichomotomized age, education and income into high (above median value) and low (at or below median value) categories. Place of residence was categorized into urban (city setting) and nonurban (town or rural setting).

Construct validity can be established by determining whether a given measure is associated with a measure representing an analogous concept. Guided by previous assessments of the validity of the Reproductive Autonomy Scale, we determined that engaging in unprotected sex in the last month represented an analogous concept to reproductive autonomy within this population of women who did not desire pregnancy in the next 12 months [6]. To assess whether participants engaged in unprotected sex in the past month, women were asked to asked to report on the methods they were currently using to prevent pregnancy, as well as the frequency which they practiced these methods when they had sex in the past month (i.e., “Always,” “More than half the time,” “About half the time,” “Less than half the time,” “Never”). We classified women as engaging in unprotected sex in the past month if they did not use a modern contraceptive method (which in this study included female or male sterilization, IUDs, implants, oral contraception, injections, female or male condoms [16]) or, among those using a modern contraceptive method, if they reported not always using the method when they had sex in the past month. Accordingly, we classified women as not engaging in unprotected sex in the past month if they were currently using a modern contraceptive method and always used this method during sex in the past month. Using Pearson’s *χ*^2^ tests, we compared women who did and did not engage in unprotected sex in the past month by various demographic characteristics.

To assess internal consistency, we calculated Cronbach’s alpha scores within each subscale and for the full Reproductive Autonomy Scale. We then assessed construct validity of the Reproductive Autonomy Scale by examining the association between each subscale score on engagement in unprotected sex in the past month using Wilcoxon ranked-sum tests. Further, we used unadjusted and adjusted logistic regression models to examine the effect of each subscale on the odds of engaging in unprotected sex. Subscale scores were modeled as continuous variables. For the adjusted logistic regression analysis, we included the following confounders based on a priori hypotheses and previous research on factors affecting both contraceptive use and household or reproductive autonomy: age [6], income [17], educational attainment [6,18,19] and place of residence [20].

## 3 Results

We included all women in the parent study sample (*N* = 500) in our analysis. The mean age of participants was 34.1 years [standard deviation (SD), 5.3]. Most participants (90.0%) lived in an urban area, were married (96.6%) and identified ethnically as Kinh (95.0%). The median income was 20 million Vietnamese dong (interquartile range [IQR], 15–30 million Vietnamese dong), and most (74.4%) had attended education beyond upper secondary school (Table 1).

**Table 1:** Demographic characteristics, overall and by engagement in unprotected sex^a^ in the past month among sexually active, reproductive-age women not desiring pregnancy in Hanoi, Vietnam, 2017–2018

			Unprotected sex in the past month^a^				
	Overall (*N* = 500)		Yes (*n* = 85)		No (*n* = 415)		p value
	*n*	(%)	*n*	(%)	*n*	(%)	
Age in years							
21–31	156	(31.2)	22	(25.9)	134	(32.3)	.38
32–36	173	(34.6)	29	(34.1)	144	(34.7)	
37–45	171	(34.2)	34	(40.0)	137	(33.0)	
Residence							
Urban area	450	(90.0)	70	(82.3)	380	(91.6)	.01
Town or rural area	50	(10.0)	15	(17.7)	35	(8.4)	
Marital status							
Married	483	(96.6)	80	(94.1)	403	(97.1)	.12
Not married	16	(3.2)	5	(5.9)	11	(2.7)	
Missing	1	(0.2)	0	(0)	1	(0.2)	
Ethnicity							
Kinh	475	(95.0)	81	(95.3)	394	(94.9)	.89
Non-Kinh	25	(5.0)	4	(4.7)	21	(5.1)	
Monthly household income							
Less than 15,000,000 Vietnamese dong	98	(19.6)	23	(27.1)	75	(18.1)	.09
At least 15,000,000 Vietnamese dong	350	(70.0)	56	(65.9)	294	(70.8)	
Missing	52	(10.4)	6	(7.0)	46	(11.1)	
Highest level of education completed							
Primary or lower secondary	34	(6.8)	7	(8.2)	27	(6.5)	.23
Upper secondary	94	(18.8)	21	(24.7)	73	(17.6)	
Higher	372	(74.4)	57	(67.1)	315	(75.9)	

a Women were classified as engaging in unprotected sex in the past month if they did not use modern contraceptive methods (i.e., COCs, IUDs, injections, implants, diaphragm, female or male condoms, female or male sterilization) or, if they used modern contraceptive methods, if they reported that these methods were not always used when they had sex in the past month.

**Fig. 1:**
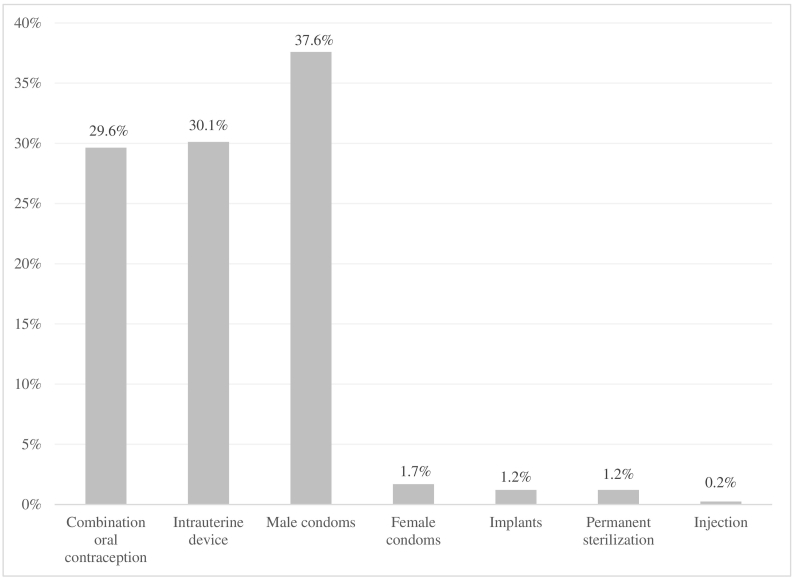
Contraceptive method use among women who did not engage in unprotected sex in the past month.

Most women (83%; *n* = 415) in our sample did not engage in unprotected sex in the past month (Table 1). A larger proportion of women who engaged in unprotected sex in the past month lived in a town or rural area relative to those who did not engage in unprotected sex (17.7% vs. 8.4%; p =.01). As compared to women who did not engage in unprotected sex in the past month, women who engaged in unprotected sex tended to be unmarried, to be less educated and to report lower household incomes, but these differences were not statistically significant at *α* = 0.05. Engaging in unprotected sex in the past month did not differ significantly by age or ethnicity. Of the women who did not engage in unprotected sex in the past month, most used combination oral contraceptives (COCs) (29.6%), and IUDs (30.1%) or male condoms (37.6%) (Fig. 1). Of the women who *did* engage in unprotected sex in the past month (*n* = 85), 27 (31.8%) used the rhythm method to prevent pregnancy, 60 (70.6%) used withdrawal, and 9 (10.6%) used no method. Nineteen (22.3%) of the women who engaged in unprotected sex reported that they currently used a modern contraceptive method but that this method was not always used when they had sex in the past month.

Findings revealed moderate internal consistency for the decision-making power subscale (Cronbach’s alpha: 0.65) and high internal consistency for the freedom from coercion and communication ability subscales (Cronbach’s alpha: 0.85 and 0.87, respectively). The full scale had a Cronbach’s alpha score of 0.58, indicating low internal consistency. The mean scores for reproductive autonomy subscales were 2.44 for decision-making power (range 0–3), 3.17 for freedom from coercion (range 0–4) and 2.17 for communication ability (range 0–4) (Table 2).

**Table 2:** Subscale scores of the Reproductive Autonomy Scale by engagement in unprotected sex in the past month among sexually active, reproductive-age women not desiring pregnancy in Hanoi, Vietnam, 2017–2018

			Unprotected sex in the past month^a^				
	Overall (*N* = 500)		Yes (*n* = 85)		No (*n* = 415)		p value^c^
Subscales^b^	Mean (SD)		Mean (SD)		Mean (SD)		
Decision making^d^	2.44	(0.54)	2.39	(0.54)	2.45	(0.54)	.26
Freedom from coercion^e^	3.17	(0.43)	3.16	(0.50)	3.18	(0.42)	.46
Communication^f^	2.17	(0.38)	2.23	(0.44)	2.16	(0.37)	.17

a Women were classified as engaging in unprotected sex in the past month if they did not use modern contraceptive methods (i.e., COCs, IUDs, injections, implants, female or male condoms, female or male sterilization) or, if they used modern contraceptive methods, if they reported that these methods were not always used when they had sex in the past month.

b Higher mean values represent greater reproductive autonomy.

c Based on Wilcoxon ranked-sum tests comparing those using a COC or IUD with those using another or no method.

d *n* = 473; based on scale 1–3.

e *n* = 498; based on scale 1–4.

f *n* = 496; based on scale 1–4.

In unadjusted logistic regression analysis, none of the reproductive autonomy subscales led to statistically significantly greater odds of unprotected sex in the past month. Decision-making power subscale scores were lower among women who engaged in unprotected sex in the past month as compared to women who did not engage in unprotected sex; however, this difference was not statistically significant (2.39 vs. 2.45, respectively; p =.26; Table 2). Women who engaged in unprotected sex in the past month also exhibited higher mean subscale scores for communication ability, but these differences, too, were not statistically significant (2.23 vs. 2.16; p =.17). Finally, mean subscale scores for freedom from coercion did not vary when comparing women who did and did not engage in unprotected sex in the past month (3.16 vs. 3.18; p =.46). Our results were similar for all subscales and outcomes after adjusting for age, income, education and residence (Table 3).

**Table 3:** Association between subscale scores of the Reproductive Autonomy Scale and engagement in unprotected sex in the past month^a^ among sexually active, reproductive-age women not desiring pregnancy in Hanoi, Vietnam, 2017–2018, *N* = 500

	Unadjusted analysis		Adjusted analysis^c^	
Subscales^b^	Odds ratio	(95% CI)	Odds ratio	(95% CI)
Decision making	0.79	(0.87–2.78)	0.77	(0.49–1.20)
Freedom from coercion	0.90	(0.52–1.55)	0.94	(0.52–1.67)
Communication	1.56	(0.87–2.78)	1.69	(0.92–3.09)

CI, confidence interval.

a Women were classified as engaging in unprotected sex in the past month if they did not use modern contraceptive methods (i.e., COCs, IUDs, injections, implants, female or male condoms, female or male sterilization) or, if they used modern contraceptive methods, if they reported that these methods were not always used when they had sex in the past month.

b Higher values represent greater reproductive autonomy.

c Adjusted for age, education, income and residence.

## 4 Discussion

In this study of reproductive-age, sexually active women not desiring pregnancy in Hanoi, Vietnam, we found that variations in reproductive autonomy, as measured using the Reproductive Autonomy Scale, did not correspond to differences in engagement in sex unprotected by a modern contraceptive method in the past month. These findings do not align with those of previous studies that examined reproductive autonomy using the Reproductive Autonomy Scale in populations within the United States: when assessing the validity of the scale among women attending family planning and abortion clinics, Upadhyay et al. found that freedom from coercion and communication ability subscales were inversely associated with unprotected sex in the past 3 months [6]. Similarly, a study of young adults in a rural university population in the United States revealed that mean scores for the communication subscale were significantly associated with use of condoms or oral contraception [20].

The reasons why construct validity was observed in the previous study by Upadhyay et al., but not in the present study, are unknown. The differences between the two study populations are vast, given the disparate cultural, economic and social contexts where the two studies were conducted. Although both studies recruited women from healthcare facilities, Upadhyay et al. recruited women from several family planning and abortion clinics throughout the United States, while our study recruited women from one obstetrics-gynecology department in Hanoi, Vietnam. Furthermore, the earlier study included a demographically diverse sample of women in the United States who were largely unmarried, whereas our study sample was comprised almost uniformly of educated, married women of a single ethnicity. For example, it could be that among women with a certain level of education in Vietnam, variations in reproductive autonomy do not influence contraception use. Notably, the mean scores for all subscales of reproductive autonomy were lower in our study population relative to those of the earlier study, which reported mean subscale scores of 3.57, 3.53 and 2.46 for freedom from coercion, communication and decision making, respectively. Our mean communication subscale score was markedly lower than that of the population in the earlier study (2.17 vs. 3.53, respectively), which may reflect differences in cultural norms or gender dynamics between these settings.

Further, our study examined the effect of the reproductive autonomy subscales on the engagement in unprotected sex within the past month, whereas Upadhyay et al. examined their effects on engagement in unprotected sex within the past 3 months [6]. We cannot rule out the possibility that our results could be different had we assessed unprotected sex in the past 3 months rather than 1 month. Nonetheless, our present outcome, although not exactly aligned with that of the earlier study, should be sufficiently related to reproductive autonomy to assess construct validity (i.e., the extent to which the scale — or subscales, in this case — are associated with measures representing analogous concept).

The lack of correspondence between low mean subscale scores and contraceptive use could indicate that the Reproductive Autonomy Scale did not capture other influential components of reproductive autonomy in this population, such as family and community influences or consistent access to care. Women also could be employing strategies to subvert their partner’s will, or communicating or negotiating through indirect means (e.g., nonverbal communication). Additionally, although some women may exhibit higher reproductive autonomy, they may lack control over other aspects of their lives that could indirectly influence reproductive decisions; studies assessing women’s general autonomy have demonstrated that women’s household decision-making autonomy increases the likelihood that a woman receives antenatal and delivery care [17] and decreases the likelihood of unintended pregnancy [21]. Finally, measures of reproductive autonomy in Vietnam might need to account for important context-specific cultural factors, such as perceived importance of social norms regarding pregnancy following marriage, expected degree of the woman’s subordination to her husband or partner’s extended family and the woman’s confidence in her interactions with healthcare providers [22]. Researchers could add and test items related to these factors to develop a version of the Reproductive Autonomy Scale that is valid for Vietnam, or Southeast Asia more broadly.

The present study population was limited to sexually active women who did not desire pregnancy and, thus, arguably could be expected to be able to avoid unprotected sex if they had sufficient reproductive autonomy. However, the role of reproductive autonomy on contraceptive use also could depend upon the woman’s preferences regarding contraceptive use. For example, women could have high reproductive autonomy but choose not to use a modern method of contraception perhaps because of concerns about the side effects or, in the case of coitally dependent methods, reductions in the act’s spontaneity. Furthermore, some women could be coerced into using a method that they do not want to use. Thus, simply measuring recent unprotected sex is a crude method of establishing the scale’s construct validity. Future research could take into account women’s preferences regarding contraception use, in general, and choice of specific methods.

Our study population was relatively homogenous, with almost all participants consisting of married, urban women of Kinh ethnicity. Respondents also had a higher overall educational status compared to women in Vietnam nationally. About 96% of respondents had completed upper secondary or higher; in comparison, only 23%–44% of women 20–45 years of age in Vietnam in 2009 had completed this level [23]. Thus, the generalizability of our findings may be limited to similar population groups. On the other hand, the homogeneity of the study population would reduce the likelihood that differences by method use were due to unmeasured confounders. A second limitation is the potential for selection bias given that women recruited from an obstetrics-gynecology department in Hanoi may have greater reproductive autonomy relative to other Vietnamese women of reproductive age. Selecting on exposure status (i.e., reproductive autonomy) could distort the relationship between exposure and outcome (i.e., contraceptive use) if results were generalized to all reproductive-age women within Vietnam.

Despite these limitations, our study is the first to assess the validity of the Reproductive Autonomy Scale in a population outside of the United States. Assessing reproductive autonomy and other factors contributing to contraceptive use is especially critical in low- and middle-income countries, as these regions experience disproportionate rates of unintended pregnancy [1]. According to data from the United Nations Population Fund, approximately 77% of women with contraceptive need are using modern contraceptive methods in Vietnam [24]. Nonetheless, despite the high reported use of contraception, the rate of abortion in Vietnam is among the highest in the world [25,26]. This anomaly of exhibiting both high rates of contraceptive use and abortion may be attributed to inconsistent contraceptive use, discontinuation or use of less effective methods. As limited reproductive autonomy may contribute to the wide gap between the availability of effective contraceptive methods and their use — in Vietnam as well as other low- or middle-income settings v researchers should work to develop and validate a measure for reproductive autonomy that can be used for populations outside the United States or to adapt the existing measure for these contexts.

## Declarations of interest

None.
